# Supplementary material for: AePUb promoter length modulates gene expression in Aedes aegypti
Source: Sci Rep. 2023 Nov 21;13:20352. doi: 10.1038/s41598-023-47777-3 (PMC10663553; doi:10.1038/s41598-023-47777-3)
Supplement: Supplementary file 1 — Supplementary Information. [file 41598_2023_47777_MOESM1_ESM.pdf]

AePUb promoter length modulates gene expression in *Aedes aegypti*

Michelle A.E. Anderson[*1,2], Philip T. Leftwich[1,3], Ray Wilson[1,2], Leonela Z. Carabajal Paladino[1], Sanjay Basu[1,4], Sara Rooney[1,5], Zach N. Adelman[6], Luke Alphey[*1,2]

[1]Arthropod Genetics, The Pirbright Institute, Ash Road, Pirbright, GU24 0HN, U.K.

[2]Department of Biology, University of York, Heslington, YO10 5DD, U.K.

[3]Current address: School of Biological Sciences, University of East Anglia, Norwich, NR4 7TJ, U.K.

[4]Current address: Molecular Biology Team, R&D Division, Oxitec, Oxford, UK.

[5]Current address: Department of Vector Biology and Department of Tropical Disease Biology, Liverpool School of Tropical Medicine, Liverpool, UK.

[6]Department of Entomology, Texas A&M University, College Station, Texas, U.S.A.

[*]Correspondence to: michelle.anderson@york.ac.uk, luke.alphey@york.ac.uk

Supplementary Table 1. Fixed and random effects table for the generalized linear mixed model (GLMM) fitted to the Luciferase Ratio detected in the engineered *PUb* promoter truncations.

	Values		
Predictors	*Estimates*	*CI*	*p*
**(Intercept)**	95.65	76.44 – 119.70	**<0.001**
**Promoter-565**	0.91	0.79 – 1.04	0.158
**Promoter-465**	0.22	0.18 – 0.27	**<0.001**
**Promoter-365**	0.74	0.64 – 0.85	**<0.001**
**Promoter-265**	0.61	0.53 – 0.71	**<0.001**
**Promoter-133**	0.61	0.52 – 0.70	**<0.001**
**Promoter-133 (77bp intron)**	0.03	0.02 – 0.04	**<0.001**
**Promoter [no FF]**	0.00	0.00 – Inf	0.991
**cell line [C636]**	0.61	0.47 – 0.79	**<0.001**
**cell line [u4.4]**	0.81	0.67 – 0.97	**0.022**
**Promoter-565:cell_lineC636**	1.22	0.86 – 1.74	0.266
**Promoter-465:cell_lineC636**	4.68	3.18 – 6.89	**<0.001**
**Promoter-365:cell_lineC636**	1.20	0.83 – 1.74	0.327
**Promoter-265:cell_lineC636**	1.71	1.19 – 2.45	**0.004**
**Promoter-133:cell_lineC636**	1.21	0.82 – 1.78	0.333
**Promoter-133 (77bp intron):cell_lineC636**	1.13	0.46 – 2.78	0.796
**Promoter [no FF] × cell line [C636]**	0.70	0.00 – Inf	1.000
**Promoter-565:cell_lineu4.4**	0.83	0.65 – 1.06	0.127
**Promoter-465:cell_lineu4.4**	1.31	0.95 – 1.82	0.102
**Promoter-365:cell_lineu4.4**	0.86	0.67 – 1.11	0.237
**Promoter-265:cell_lineu4.4**	0.99	0.76 – 1.28	0.914
**Promoter-133:cell_lineu4.4**	0.78	0.60 – 1.02	0.075
**Promoter-133 (77bp intron):cell_lineu4.4**	0.06	0.01 – 0.42	**0.005**
**Promoter [no FF] × cell line [u4.4]**	0.40	0.00 – Inf	1.000
**Random Effects**			
**σ^2**	0.27		
**$\tau_{00\ experiment}$**	0.10		
**ICC**	0.28		
**$N_{experiment}$**	10		
**N_{obs}**	941		
**Observations**	941		
**Marginal R^2 / Conditional R^2**	0.993 / 0.995		

Supplementary Table 2. TFsitescan results.

Site (Length)	Position relative to TSS(0)	Score (Gaps)	Occurrence	EA Probability	Notes
**PEA3-TBXAS1-3'(8)**	-2157	8 (0)	0	1.80E-02	
**Genesis-site-25(12)**	-2054	11 (0)	0	3.16E-01	
**C/EBP-LTR(1)'(9)**	-2048	9 (0)	0	4.49E-03	
**E4BP4_CS'(8)**	-2048	7 (0)	0	7.18E-02	
**E4BP4_CS(8)**	-2048	7 (0)	0	7.18E-02	
**C/EBP-beta_CS(9)**	-2048	7 (0)	0	2.87E-01	
**ALAS2-GATA-A'(8)**	-2027	8 (0)	0	1.80E-02	
**E2F-muCdc7-2'(9)**	-2019	9 (0)	0	4.49E-03	
**HIP1-DHFR(13)**	-2018	12 (0)	0	7.89E-02	
**E2F4/DP_consensus(9)**	-2017	8 (0)	0	1.80E-02	
**E2F-DHFR(12)**	-2017	11 (0)	0	3.16E-01	
**EIIF-EIIaE1'(8)**	-2017	8 (0)	0	1.80E-02	
**E2F_CS.1(8)**	-2017	8 (0)	0	1.80E-02	
**NF-mu-E1_CS'(8)**	-2011	7 (0)	0	7.18E-02	
**E2F-c-Myc-site-II(8)**	-1924	8 (0)	0	1.80E-02	
**IRF-2_RS(8)**	-1867	6 (0)	0	2.87E-01	
**90K-IRF-E(12)**	-1863	11 (0)	0	3.16E-01	
**pdx1-Ph1-site-1'(11)**	-1847	10 (0)	0	1.00E+00	
**HSF-consensus(15)**	-1845	10 (0)	0	1.00E+00	
**HSF-consensus'(15)**	-1840	10 (0)	0	1.00E+00	
**HSF-consensus(15)**	-1840	10 (0)	0	1.00E+00	
**HSF-consensus'(15)**	-1835	10 (0)	0	1.00E+00	
**HSF-consensus(15)**	-1835	10 (0)	0	1.00E+00	
**TTF-1-CAM-III-B'(10)**	-1804	10 (0)	0	1.12E-03	
**C/EBP-beta_CS(9)**	-1801	7 (0)	0	2.87E-01	
**MIG-pIRE'(11)**	-1776	10 (0)	0	1.00E+00	
**hH3/m-m8-TATA(9)**	-1772	9 (0)	0	4.49E-03	
**PRL_conserved_motif**					
**beta-RARE-NBRE(8)**	-1659	8 (0)	0	1.80E-02	
**EKLF_CS(9)**	-1621	7 (0)	0	2.87E-01	
**OVO_CS2(8)**	-1613	7 (0)	0	7.18E-02	
**HSF-consensus'(15)**	-1595	10 (0)	0	1.00E+00	
**TIE_(2)(10)**	-1576	8 (0)	0	1.80E-02	
**Mt3(8)**	-1484	8 (0)	0	1.80E-02	
**CNTF_response_eleme**					
**CNTF_response_eleme**					
**MEF-2_CS3'(9)**	-1457	7 (0)	0	7.18E-02	
**PSTI-Region-IV-core(11)**	-1445	10 (0)	0	1.00E+00	
**HNF1-HP1'(13)**	-1445	9 (0)	0	7.18E-02	
**Brn3b_CS(9)**	-1443	7 (0)	0	7.18E-02	

**CNTF_response_eleme**				
**PEA-3-TBXAS1'(8)**	-1406	8 (0)	0	1.80E-02
**DHFR-undefined-site**				
**TTF-TPO-2'(8)**	-1365	8 (0)	0	1.80E-02
**Rh50-Ets-1(8)**	-1360	8 (0)	0	1.80E-02
**PR-uterogl.2(8)**	-1360	8 (0)	0	1.80E-02
**optimal-Inr-sequenc**				
**HBP1-N-myc-1(8)**	-1344	8 (0)	0	1.80E-02
**YY1_CS4'(11)**	-1338	9 (0)	0	1.00E+00
**Ren-1c-CRE(12)**	-1333	11 (0)	0	3.16E-01
**TTF-TPO-2'(8)**	-1310	8 (0)	0	1.80E-02
**SREBP-1_CS'(9)**	-1289	7 (0)	0	1.44E-01
**GT-IIC-SV40(11)**	-1257	10 (0)	0	1.00E+00
**CNTF_response_eleme**				
**CNTF_response_eleme**				
**C/EBP-SV40-1(8)**	-1254	8 (0)	0	1.80E-02
**IRF-2_RS'(8)**	-1237	6 (0)	0	2.87E-01
**YY1-involucrin-1(11)**	-1237	10 (0)	0	1.00E+00
**CNTF_response_eleme**				
**c-Ets-2_CS'(8)**	-1228	7 (0)	0	7.18E-02
**HSTF_CS3'(15)**	-1209	10 (0)	0	1.00E+00
**kappaY'(8)**	-1122	8 (0)	0	1.80E-02
**HS-40_GATA1(d)'(11)**	-1105	10 (0)	0	1.00E+00
**eHTF-albumin'(9)**	-1080	9 (0)	0	4.49E-03
**BETA2/NeuroD-NNAT-1(12)**	-1076	11 (0)	0	3.16E-01
**YY1_CS(9)**	-1066	7 (0)	0	1.44E-01
**IgHC.20(8)**	-1066	8 (0)	0	1.80E-02
**CuE1.1(8)**	-1066	8 (0)	0	1.80E-02
**CuE1.2(8)**	-1064	8 (0)	0	1.80E-02
**Math3-E-box'(8)**	-1017	8 (0)	0	1.80E-02
**T3R_TRE1_CS(8)**	-839	7 (0)	0	7.18E-02
**Ets1-erk1(8)**	-837	8 (0)	0	1.80E-02
**MyoD_CS2(9)**	-786	7 (0)	0	1.44E-01
**HiNF-A_RS(12)**	-640	9 (0)	0	1.80E-02
**HSF-consensus(15)**	-591	10 (0)	0	1.00E+00
**epsilon-globin-unde**				
**HNF1-HP1'(13)**	-534	9 (0)	0	7.18E-02
**STAT5A_CS'(9)**	-486	7 (0)	0	7.18E-02
**STAT5A_CS(9)**	-486	7 (0)	0	7.18E-02
**CNTF_response_eleme**				
**TB1'(9)**	-486	9 (0)	0	4.49E-03
**beta-pol_CS(10)**	-458	8 (0)	0	1.80E-02
**beta-pol_CS'(10)**	-458	8 (0)	0	1.80E-02
**ATF3_CS'(8)**	-457	7 (0)	0	7.18E-02
**ATF3_CS(8)**	-457	7 (0)	0	7.18E-02

**CREB_CS2'(8)**	-457	7 (0)	0	7.18E-02	
**CREB_CS2(8)**	-457	7 (0)	0	7.18E-02	
**CRE-som'(8)**	-457	8 (0)	0	1.80E-02	
**CRE-som(8)**	-457	8 (0)	0	1.80E-02	
**CRE-Ga'(8)**	-457	8 (0)	0	1.80E-02	
**CRE-Ga(8)**	-457	8 (0)	0	1.80E-02	
**TCR-beta_decamer'(10)**	-457	9 (0)	0	4.49E-03	
**b2-somatostatin'(8)**	-457	8 (0)	0	1.80E-02	
**b2-somatostatin(8)**	-457	8 (0)	0	1.80E-02	
**cAMP_RE'(8)**	-457	8 (0)	0	1.80E-02	
**cAMP_RE(8)**	-457	8 (0)	0	1.80E-02	
**TRAC_CS'(8)**	-454	7 (0)	0	3.59E-02	
**PEBP5-A_enhancer'(11)**	-453	10 (0)	0	1.00E+00	
**Ets-1-Octa-1'(8)**	-430	8 (0)	0	1.80E-02	
**IRF-2_RS(8)**	-403	6 (0)	0	2.87E-01	
**HiNF-A_RS'(12)**	-398	9 (0)	0	1.80E-02	
**E2A_US'(8)**	-397	8 (0)	0	1.80E-02	
**C/EBP-beta_CS'(9)**	-396	7 (0)	0	2.87E-01	
**Six5_CS(9)**	-360	7 (0)	0	7.18E-02	
**GATA-1-beta-globin_((8)**	-341	8 (0)	0	1.80E-02	
**HiNF-A_RS'(12)**	-315	9 (0)	0	1.80E-02	
**c-Ets-2_CS'(8)**	-311	7 (0)	0	7.18E-02	
**delta_repressing_el**					
**ssT1_CS(12)**	-283	8 (0)	0	1.44E-01	
**PEA-3-TBXAS1(8)**	-273	8 (0)	0	1.80E-02	
**CREB/ATF-c-jun'(8)**	-234	8 (0)	0	1.80E-02	
**CREB_CS2(8)**	-234	7 (0)	0	7.18E-02	
**CREB-HTLVI.1(8)**	-234	8 (0)	0	1.80E-02	
**TFII-I-40/fos(8)**	-156	8 (0)	0	1.80E-02	
**delta_repressing_el**					
**IL-4-MARE'(9)**	-77	9 (0)	0	4.49E-03	
**C/EBP-beta_CS'(9)**	-77	7 (0)	0	2.87E-01	
**HSE_CS_inverted_rep**					
**HSE_CS_inverted_rep**					
**PAUSE-1_CS1'(13)**	-71	10 (0)	0	1.00E+00	
**HSTF_CS3(15)**	-71	10 (0)	0	1.00E+00	
**GR/PR-uteroglobin.1**					
**PR-uterogl.3'(8)**	23	8 (0)	0	1.80E-02	intron
**HSF-consensus(15)**	59	10 (0)	0	1.00E+00	intron
**S8_site(11)**	93	9 (0)	0	1.00E+00	intron
**ICS-MTII'(12)**	105	11 (0)	0	3.16E-01	intron
**ICS-HLADR'(12)**	105	11 (0)	0	3.16E-01	intron
**ICS_(1)'(12)**	105	11 (0)	0	3.16E-01	intron

**GR/PR-uteroglobin.1**					intron
**PR-uterogl.3'(8)**	122	8 (0)	0	1.80E-02	intron
**LEF1_CS'(8)**	138	7 (0)	0	3.59E-02	intron
**Rh50-Ets-2'(8)**	141	8 (0)	0	1.80E-02	intron
**LHX4-POU1F1(8)**	211	8 (0)	0	1.80E-02	intron
**EIIaE-B''(8)**	237	8 (0)	0	1.80E-02	intron
**LEF1_CS(8)**	268	7 (0)	0	3.59E-02	intron
**delta_repressing_el**					intron
**YY1_CS2'(9)**	292	8 (0)	0	1.80E-02	intron
**YY1_CS(9)**	293	7 (0)	0	1.44E-01	intron
**IgHC.20(8)**	293	8 (0)	0	1.80E-02	intron
**CuE1.1(8)**	293	8 (0)	0	1.80E-02	intron
**CD14-TLS(8)**	371	8 (0)	0	1.80E-02	intron
**paired_CS2'(11)**	412	9 (0)	0	1.80E-02	intron
**paired_CS2(11)**	412	9 (0)	0	1.80E-02	intron
**HSTF_CS3(15)**	420	10 (0)	0	1.00E+00	intron
**ARE_CS'(10)**	453	7 (0)	0	1.44E-01	intron
**TRAC_CS'(8)**	457	7 (0)	0	3.59E-02	intron
**MEF-2_CS3'(9)**	467	7 (0)	0	7.18E-02	intron
**FKHRL1-FasL(11)**	468	10 (0)	0	1.00E+00	intron
**UL127-WH-site'(11)**	470	10 (0)	0	1.00E+00	intron
**E2_RS1'(12)**	493	8 (0)	0	2.87E-01	intron
**E2_RS1(12)**	493	8 (0)	0	2.87E-01	intron
**BPV-E2_CS2'(12)**	493	8 (0)	0	2.87E-01	intron
**BPV-E2_CS2(12)**	493	8 (0)	0	2.87E-01	intron
**Pdx-1-insulin-P1'(8)**	496	8 (0)	0	1.80E-02	intron
**C/EBP-beta_CS'(9)**	563	7 (0)	0	2.87E-01	intron
**Cited2-SRE(11)**	604	10 (0)	0	1.00E+00	intron
**IRF-2_RS'(8)**	604	6 (0)	0	2.87E-01	intron
**beta-pol_CS(10)**	629	8 (0)	0	1.80E-02	intron
**ATF_CS1(9)**	629	8 (0)	0	1.80E-02	intron
**beta-pol_CS'(10)**	629	8 (0)	0	1.80E-02	intron
**ATF3_CS'(8)**	630	7 (0)	0	7.18E-02	intron
**ATF3_CS(8)**	630	7 (0)	0	7.18E-02	intron
**CREB_CS2'(8)**	630	7 (0)	0	7.18E-02	intron
**CREB_CS2'(8)**	630	7 (0)	0	7.18E-02	intron
**CREB_CS2(8)**	630	7 (0)	0	7.18E-02	intron
**CRE-som'(8)**	630	8 (0)	0	1.80E-02	intron
**CRE-som(8)**	630	8 (0)	0	1.80E-02	intron
**CRE-Ga'(8)**	630	8 (0)	0	1.80E-02	intron
**CRE-Ga(8)**	630	8 (0)	0	1.80E-02	intron
**b2-somatostatin'(8)**	630	8 (0)	0	1.80E-02	intron
**b2-somatostatin(8)**	630	8 (0)	0	1.80E-02	intron
**cAMP_RE'(8)**	630	8 (0)	0	1.80E-02	intron
**cAMP_RE(8)**	630	8 (0)	0	1.80E-02	intron

**rHemopexin-A-site'(11)**	631	10 (0)	0	1.00E+00	intron
**IRF-2_RS'(8)**	657	6 (0)	0	2.87E-01	intron
**CK-8-mer(8)**	726	7 (0)	0	7.18E-02	intron
**keratinocyte_CS(8)**	726	7 (0)	0	3.59E-02	intron
**AP-2_beta_CS'(9)**	728	6 (0)	0	5.75E-01	intron
**AP-2_beta_CS(9)**	728	6 (0)	0	5.75E-01	intron
**AP-2_site'(9)**	728	7 (0)	0	2.87E-01	intron
**AP-2_site(9)**	728	7 (0)	0	2.87E-01	intron
**HNF-6-r.cyp2c13(10)**	740	10 (0)	0	1.12E-03	intron
**SREBP-1_CS'(9)**	748	7 (0)	0	1.44E-01	intron
**IBP-1_CS'(14)**	758	11 (0)	0	1.00E+00	intron
**YY1-involucrin-1(11)**	760	10 (0)	0	1.00E+00	intron
**C/EBP-beta_CS'(9)**	765	7 (0)	0	2.87E-01	intron
**C/EBP-beta_CS'(9)**	777	7 (0)	0	2.87E-01	intron
**TTF-TPO-4'(8)**	780	8 (0)	0	1.80E-02	intron
**IECS'(11)**	790	9 (0)	0	1.80E-02	intron
**IRF-2_RS'(8)**	797	6 (0)	0	2.87E-01	intron

Supplementary Table 3. Primers used in this study.

Primer Number	*Primer Name*	***Sequence***	*Use*
LA6439	PUb upF	ctctatcgataggtaccgagctcttacgcgACCCGCTGCTATTCAGCAAGAGCAAGCTGA	PUb promoter F: 2196
LA6440	PUb upR	GATTCAATGCACAAGCTACATGTAAAGATATCACTTTGAGTGGTTCGTTTTTATTCAACA	PUb promoter R: 2196
LA6561	FrgA from 1447_fwd	gtcacggcgggcatgtcgacttaattaaccggccgggaggttcccac	transformation marker F: 2143-2146
LA6562	FrgA from 1447_rev	cgatcgaatatggccggccccgcccccaaacgcgcca	transformation marker R: 2143
LA6563	FrgB from 1752_fwd	gggcggggccggccatattcgatcgatatagag	PUb promoter F: 2143
LA6564	FrgB from 1752_rev	ggacagggccatggttgaaatctctgttgag	PUb promoter R: 2143-2146
LA6565	FrgC from 1037_fwd	agagatttcaaccatggccctgtccaacaagttc	AmCyan F: 2143-2146
LA6566	FrgC from 1037_rev	cgtcgggcccttaggagcggagtccggagaag	AmCyan R: 2143-2146
LA6567	FrgD from 1037_fwd	ggactccgctcctaagggcccgacgtcttc	SV40 3'UTR F: 2143-2146
LA6568	FrgD from 1037_rev	aagttatcctaggactagtggcgcgcctcattaagatacattgatgagtttgg	SV40 3'UTR R: 2143-2146
LA6571	FrgA from 1447_rev	cgtaagagctcggccggccccgcccccaaacgcgcca	transformation marker R: 2145
LA6572	FrgB from 1747_fwd	gggcggggccggccgagctcttacgcgtatc	PUb promoter F: 2145
LA6573	FrgA from 1447_rev	cgtaagagctcggccggccccgcccccaaacgcgcca	transformation marker R: 2146
LA6574	FrgB from -2565_fwd	gggcggggccggccgagctcttacgcgacc	PUb promoter F: 2146
LA6945	FrgB from 2150	cggggccggccTATGCCATATACACGAAg	PUb promoter F: 2144
LA6946	FrgA from 1447	TATGGCATAggccggccccgcccccaaacgcgcca	transformation marker R: 2144
LA3873	attL-F	TTTATCGAATTGCTTCGGCGCCAAGTAGTG	Confirmation of insertion - attL
LA7385	attL-R	CGTCGCCGTCCAGCTCGACCA	Confirmation of insertion - attL
LA7384	attR-F	CGGATAACAATTTCACACAG	Confirmation of insertion - attR
LA5816	attR-R	cgtggtacgtatacgtgtcggccgctggaaagcgaggt	Confirmation of insertion - attR

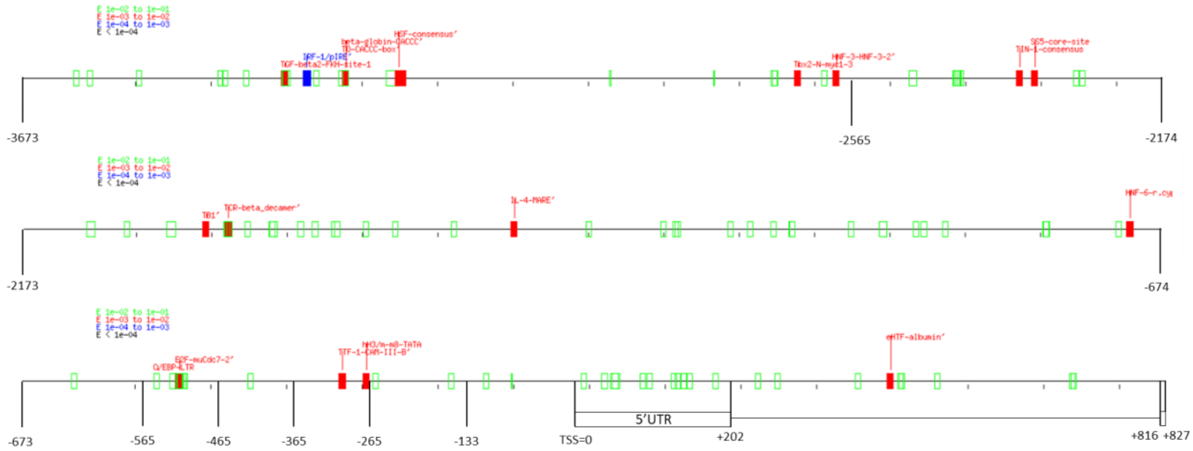

Supplementary Figure S1. Schematic of TFsite scan results. Visual output from TFsitescan with the nucleotides and promoter fragment locations indicated below the axis.

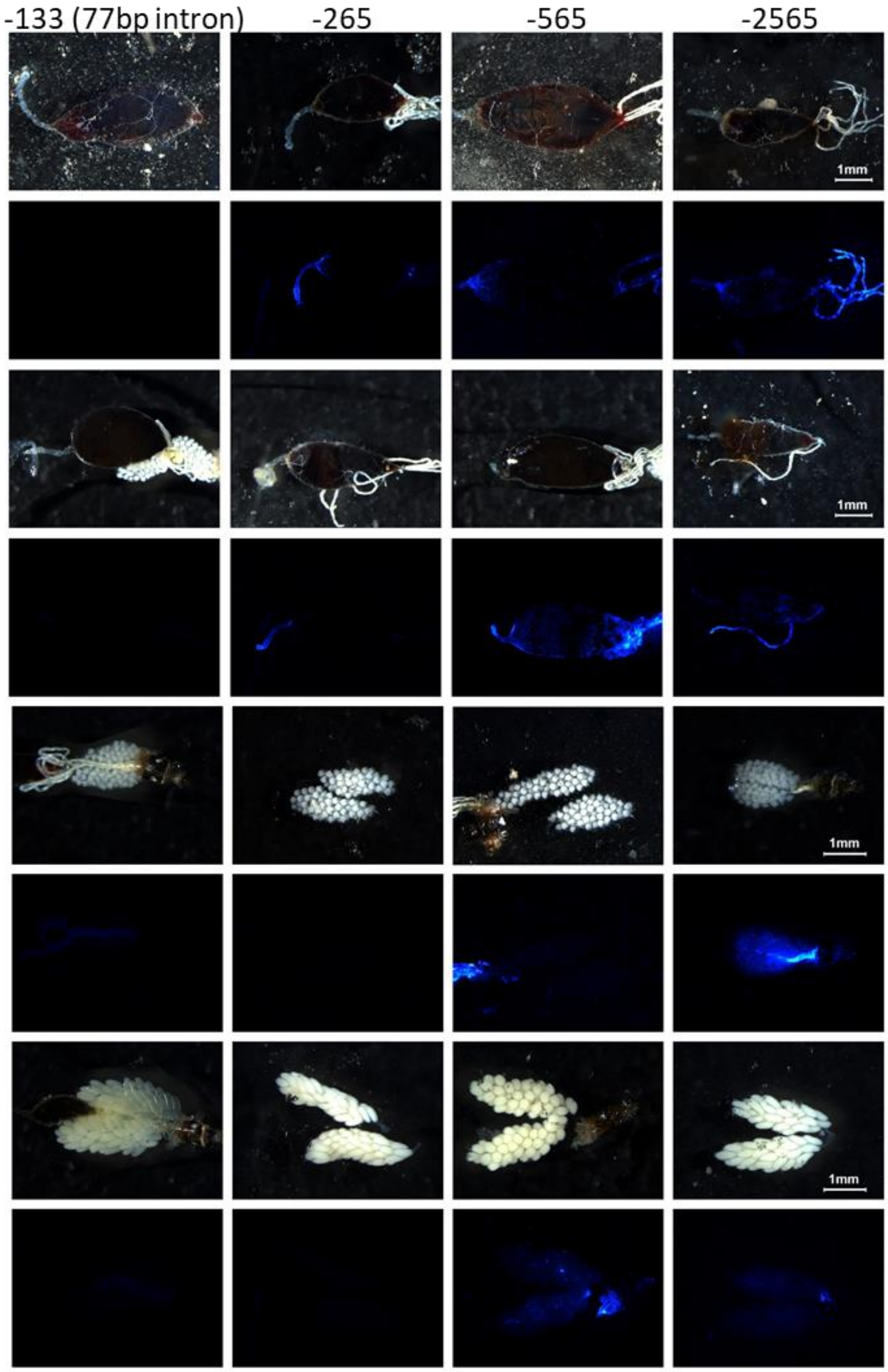

Supplementary Figure S2. ***PUb*** **promoter expression across tissues post-bloodmeal.** Brightfield and AmCyan fluorescence of female dissected gut and ovaries, 24h (top two rows, 5th and 6th rows) and 48h (3rd and 4th row, 7th and 8th rows) post-blood meal.
